# SweHLA: the high confidence HLA typing bio-resource drawn from 1000 Swedish genomes

**DOI:** 10.1038/s41431-019-0559-2

**Published:** 2019-12-16

**Authors:** Jessika Nordin, Adam Ameur, Kerstin Lindblad-Toh, Ulf Gyllensten, Jennifer R. S. Meadows

**Affiliations:** 1 Science for Life Laboratory, Department of Medical Biochemistry and Microbiology, Uppsala University, Uppsala, Sweden; 2 Science for Life Laboratory, Department of Immunology, Genetics and Pathology, Uppsala University, Uppsala, Sweden; 3 Broad Institute of MIT and Harvard, Cambridge, MA USA

**Keywords:** Immunogenetics, Computational biology and bioinformatics, Immunogenetics

## Abstract

There is a need to accurately call human leukocyte antigen (HLA) genes from existing short-read sequencing data, however there is no single solution that matches the gold standard of Sanger sequenced lab typing. Here we aimed to combine results from available software programs, minimizing the biases of applied algorithm and HLA reference. The result is a robust HLA population resource for the published 1000 Swedish genomes, and a framework for future HLA interrogation. HLA 2nd-field alleles were called using four imputation and inference methods for the classical eight genes (class I: *HLA-A*, *HLA-B*, *HLA-C*; class II: *HLA-DPA1*, *HLA-DPB1*, *HLA-DQA1*, *HLA-DQB1*, *HLA-DRB1*). A high confidence population set (SweHLA) was determined using an *n−1* concordance rule for class I (four software) and class II (three software) alleles. Results were compared across populations and individual programs benchmarked to SweHLA. Per gene, 875 to 988 of the 1000 samples were genotyped in SweHLA; 920 samples had at least seven loci called. While a small fraction of reference alleles were common to all software (class I = 1.9% and class II = 4.1%), this did not affect the overall call rate. Gene-level concordance was high compared to European populations (>0.83%), with COX and PGF the dominant SweHLA haplotypes. We noted that 15/18 discordant alleles (delta allele frequency >2) were previously reported as disease-associated. These differences could in part explain across-study genetic replication failures, reinforcing the need to use multiple software solutions. SweHLA demonstrates a way to use existing NGS data to generate a population resource agnostic to individual HLA software biases.

## Introduction

The human major histocompatibility complex (MHC) spans approximately four Mb on chromosome six and contains more than 200 genes, ~40% of which have immunological function [1]. Within this region, the human leukocyte antigen (HLA) genes are divided into classes (class I, present intracellular derived peptides to CD8+ T-cells; class II, present extracellular derived peptides to CD4+ T-cells). These are some of the most polymorphic genes in the genome, with new alleles continuously being discovered [1–3]. In the ten years from 2008 to 2018, the number of alleles in class I and class II have expanded six fold; from ~2500 to ~15,500 and ~1000 to ~6000 alleles, respectively [4]. Given their roles in immune recognition, these genes are essential to the processes of transplantation, disease and infection susceptibility (including immunological diseases, but also cancers and neuropathies), drug response and pregnancy [2, 3, 5, 6].

Sanger sequenced lab typing is the gold standard for calling HLA alleles, where alleles are usually called at the clinically relevant, protein level, 2nd-field resolution [7, 8] (e.g., HLA-A*24:02 where the fields are separated by a colon). However, the last ten years has seen the growing need to accurately call alleles from pre-existing data, such as that generated from SNP chips or NGS short-read sequencing [7]. The result has been an explosion of HLA software solutions, each using different methods for imputation or inference. The continued growth in this bioinformatics field neatly illustrates the difficulty of the task, and demonstrates how, as yet, no single software can replace biological typing.

Using four freely available software programs, and existing Illumina short read NGS data generated for the 1000 Swedish genomes project (SweGen [9]), we called 2nd-field alleles for the classical eight HLA genes (class I: *HLA-A*, *HLA-B*, *HLA-C*; class II: *HLA-DPA1*, *HLA-DPB1*, *HLA-DQA1*, *HLA-DQB1*, *HLA-DRB1*). This multi-software data set demonstrated how biases inherent in input data choice, HLA allele reference availability and software algorithms, could impact downstream analyses. For these reasons, alleles within the high confidence Swedish population HLA set, SweHLA, were designated on the basis of *n−1* software matches (class I: three out of four; class II: two out of three). This resource, benchmarked with allele frequency correlation to 252 previously lab typed Swedish individuals [10] and compared on a population level to 5544 imputed British individuals [11], is publicly available for research use.

## Methods

### Study population

Individual BAM and gVCF files from the published whole genome sequencing project of 1000 individuals, SweGen [9], were used as the basis for these analyses. Representing a cross-section of the Swedish population, these individuals were selected from the Swedish twin registry (one per pair) and The Northern Sweden Population Health Study. In total this encompassed 506 males and 494 females with a median age of 65.2 years [9]. SweGen [9] data had an average genome coverage of 36.7x and was generated using paired-end sequencing (150 bp read length) on Illumina HiSeq X with v2.5 sequencing chemistry (10.17044/NBIS/G000003).

### MHC demographics

The MHC region was defined as spanning hg19 chr6:28 477 797-33 448 354 using coordinates lifted from GRCh38.p13. Nucleotide diversity (Pi), Tajima’s D, and SNP and indel densities were calculated in 1000 bp windows from curated vcfs using VCFtools [12] v0.1.14. Coverage across the same windows was determined with BEDtools [13] v2.26.0 using individual sorted BAM files and a read length of 150 bp [9].

### HLA typing with four software

**Fig. 1:**
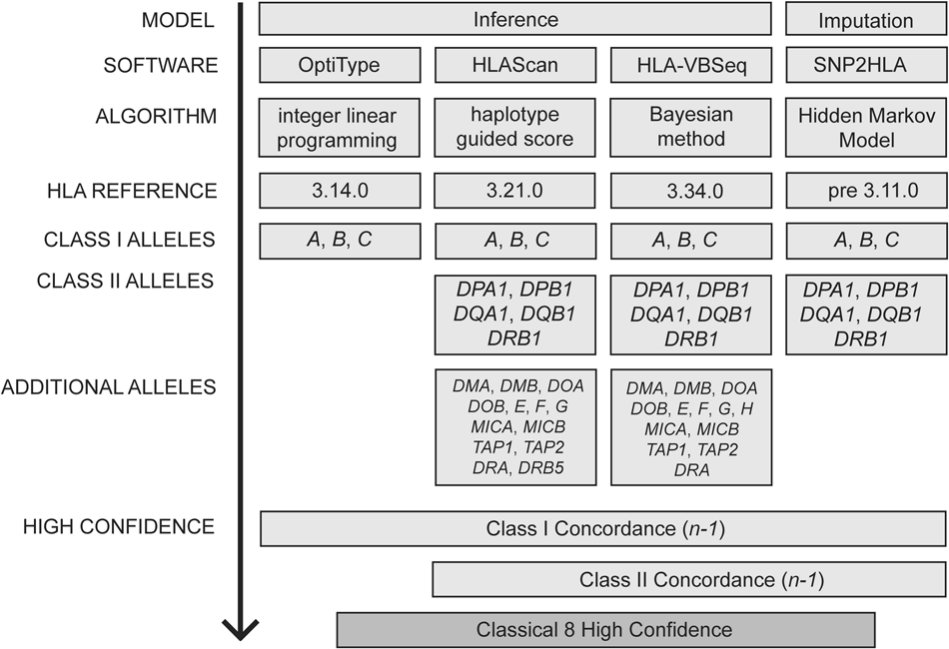
Pipeline for preliminary HLA allele typing and generation of a high confidence gene set. Four software solutions, representing two different models (imputation or inference) were used for typing. Each software utilized a separate algorithm and HLA reference to call a variable number of genes. An *n−1* concordance rule was used to create the high confidence set for the classical 8 genes (*HLA-A, HLA-B, HLA-C, HLA-DPA1, HLA-DPB1, HLA-DQA1, HLA-DQB1, HLA-DRB1*).

Four freely available software programs were selected for the analysis (Fig. 1); the commonly used imputation (SNP2HLA [14], cited >340 times) and inference software (OptiType [15], cited >140 times), as well as two more recently published inference solutions (HLA-VBSeq [16] and HLAscan [17]). In brief, the imputation method builds HLA alleles based on haplotypes generated from user supplied pruned GWAS SNPs and a phased reference panel. Whereas inference software aligns NGS reads to all HLA alleles in a reference and determines an allele best match via method specific penalty algorithms. The reference is sourced from the ImMunoGeneTics project/human leukocyte antigen (IMGT/HLA) database [18]. Of note, each software method uses a different reference version, and different regions of this resource, be it nucleotide (exonic) or genomic sequence. The 2nd-field resolution alleles from each program were recorded for each HLA gene available, however only alleles from the classical 8 genes were evaluated for the generation of SweHLA. The specific running conditions of each software program is detailed below and summarized in Fig. 1.

SNP2HLA [14] utilizes the Hidden Markov model of Beagle [19] and the T1DGC reference panel of 5225 Europeans [14] to impute HLA alleles based on a pre 3.11.0 reference (version not specified). The result is 2nd-field allele information for the classical 8 genes. The default settings, ten iterations and window size of 1000 markers, were implemented.

OptiType [15] views HLA typing as an optimization problem and uses an integer linear program to estimate which allele explains the largest number of reads. This software implements a custom-made IMGT/HLA v3.14.0 reference, where nucleotide sequences have been complemented with intronic information from the closest neighbor of the allele. These genomic-like sequences focus on exons 2 and 3 and allow for the calling of *HLA-A*, *HLA-B*, and *HLA-C*, to a 2nd-field resolution.

HLA-VBSeq [16] uses a variational Bayesian approach to remap reads to a user defined IMGT/HLA genomic reference, we selected v3.34.0. Default settings were used, however the recommended allele coverage threshold (>20% of mean coverage) was relaxed to 10% in order to increase the number of alleles reported (reduced from 30 to 0.5% NA genotypes). Coverage was calculated for the 21 genes available in HLA-VBSeq using the longest transcript and Picard v1.92 *HS-metrics* (http://broadinstitute.github.io/picard/). HLA-VBSeq typed at up to a 4-field resolution [8].

HLAscan [17] realigns reads to a reference consisting of the nucleotide sequences from IMGT/HLA v3.21.0. It relies on a score function that ranks alleles based on the number of unique reads mapping to each, including a gap penalty. Alleles are reported up to a 3rd-field resolution [8], and calls are based on exon 2 and 3 for class I genes, and exon 2 for class II genes. Default settings were used; score cut-off 50, constant using ScoreFunc 20, for the 21 available genes.

### Creation of the high confidence SweHLA data set

As indicated above, each software program has its own inherent biases. To reduce the impact of these, the high confidence SweHLA allele data set was generated by merging results based on *n−1* software concordance (Fig. 1). An individual was classed as “typed” if the allelic pairs for three out of four software matched for class I, or two out of three for class II. Downstream population allele frequencies were calculated from the SweHLA data set.

All alleles are named according to IMGT/HLA references with a subset of HGVS allele notations reported in Supplementary Table S1. The HGVS nomenclature for all alleles is maintained at https://www.ebi.ac.uk/ipd/imgt/hla/allele.html.

### Phasing of HLA haplotype blocks

SweHLA alleles were used as input to estimate haplotype blocks across the classical 8 genes with PHASE v2.1.1 [20]. The -MS model [21] was run over 10,000 iterations using a thinning interval of 5 and a burn-in of 100. The model was run ten times using different seeds for each. In order to maintain phasing power but reduce computational time, the eight genes were divided into three blocks based on known recombination hotspots (1: *HLA-C* and *HLA-B*, 2: *HLA-DRB1*, *HLA-DQA1*, and *HLA-DQB1*, and 3: *HLA-DPA1* and *HLA-DPB1)* [22]. In this way the maximum number of samples per block could also be considered. Haplotypes from the three intermediate blocks were combined in the following sequence, *HLA-A* with block 1, followed by block 2 and 3. Haplotypes were named as per the International Histocompatibility Working Group (Supplementary Table S2).

### Benchmarking the HLA typing accuracy and population frequency

Across software program comparisons were performed to investigate the impact of software choice on the ability to call HLA alleles. SweHLA was assigned as the truth set and a concordance rate per allele calculated for each program. Concordance rate was defined by counting the number of times an allele was correctly called, divided by the total number of SweHLA calls for the same allele.

Within and across population comparisons were also conducted. We estimated allele calling accuracy by comparing SweHLA allele frequencies to an independent lab typed Swedish population [10]. The lab typed set consisted of 252 unrelated individuals at 2nd-field resolution for *HLA-A*, *HLA-B*, *HLA-DQA1*, *HLA-DQB1*, and *HLA-DRB1*. Correlation (*r*^2^) was calculated with *cor()* in the R v3.4 environment [23]. To place SweHLA results in the context of Europe, gene and haplotype frequencies were compared to those of a recently published SNP2HLA imputed British population (5544 individuals) [11].

## Results

### Characterization of MHC region

**Fig. 2:**
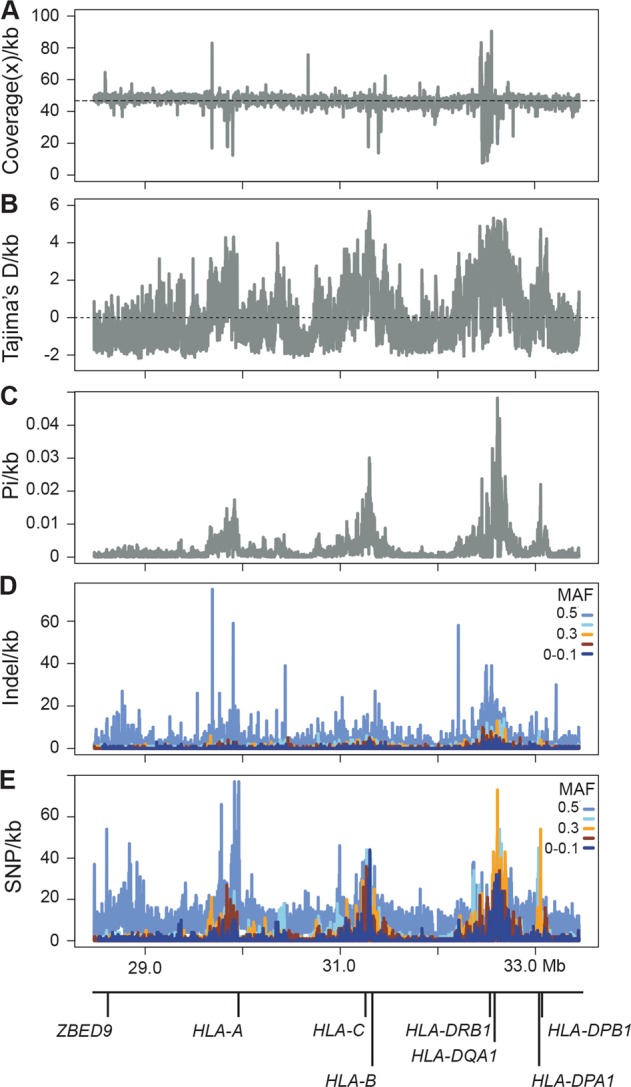
Characterization of read depth and variation in 1-kb bins across the MHC. **a** Coverage peaks and troughs are illustrated in relation to the average across the region (46.8x, dotted line). Metrics of genetic diversity, Tajima’s *D* (**b**) and Pi (**c**) are plotted for the same bins, as are density values for indels (**d**) and SNPs (**e**), although the latter are further divided into minor allele frequency (MAF) bins.

The ability to call HLA alleles across the MHC is directly related to the quantity and quality of the reads mapped and variants called. Given the variability of coverage across this four Mb region (average 46.8x; range 7.5–90.5x; Fig. 2a), we examined the repeat and gene content of the 1 kb bins sitting at the extremes of the distribution (coverage <20x or >70x). As expected, these regions predominantly contained repeat elements (61% were L1 LINEs, Alu SINEs and ERV1 LTRs), however we did note exons 1, 3, and 6 of *HLA-DRB1* (NM_002124.3 positions 1–194, 465–746, and 882–1217, respectively) were covered with >70x. These coverage extremes illustrate the inherent problems of mapping short-read data uniquely to repeats or across genes and paralogues.

We used the metrics of Tajima’s *D* and Pi, in combination with variation density, to examine the patterns of selection and diversity across the MHC (Fig. 2b–e). The three main peaks in each panel are centered over the class I (e.g., 29.9 Mb near *HLA-A* and 31.2 Mb near *HLA-C*) and class II genes (e.g., 32.6 Mb near *HLA-DQA1*), likely reflecting the selection pressure on these key immune gene classes (as Tajima’s *D* > 3, Fig. 2b). The strongest region of nucleotide diversity spanned the class II genes, with the apex including the 3′UTR of *HLA-DQA1* (NM_002122.3, Pi = 0.048, 137 SNPs; Fig. 2c). In contrast to both class I regions, the 32.6 Mb section contains the highest density of SNPs in the 0.2–0.3 and 0.4–0.5 MAF bins (orange and light blue, respectively, Fig. 2e). We further dissected the 87,637 variable positions in both the indel and SNP bins to characterize which fraction represented known (dbSNP v147) or novel variation (Supplementary Table S3). In each case, the majority of novel variation was found in the lowest MAF bin (MAF < 0.1; Supplementary Table S3, 69.0% of novel indels and 58.3% of novel SNP; Fig. 2d, e, dark blue band). For indels the fraction of novel variants per bin remained fairly steady (30–40%), however this value dropped markedly for SNPs (1.5–3.0%), and noticeably, only singletons not found in ExAC [24] variant database were located in exons 2 or 3 of the classical 8 genes (data not shown).

### HLA alleles from four software and high confidence calls

Each of the software programs applied demonstrated a high per gene HLA typing rate, ranging between 98.1–100% (Supplementary Table S4). Per software the most difficult gene to call was *HLA-DPA1* (938 samples called by HLAscan, Supplementary Table S4), while for SweHLA it was *HLA-DQA1* (824 samples typed, Table 1). The overall genotyping rate dropped slightly for SweHLA (93.7%), however this was due to cross software mismatches and not a single individual’s inability to be typed. Of the small fraction of SweHLA alleles that were called as NA (1006/16,000 alleles), most (*n* = 863) could be resolved if typing was relaxed to the serological antigen 1st-field level.

**Table 1:** Summary information for the high confidence set, SweHLA.

		Samples^1^	Alleles^2^	Homozygosity (%)	Coverage^3^
*HLA-*	*A*	981	28	17.7	37.7
	*B*	971	36	9.8	35.5
	*C*	976	23	12.1	35.1
	*DQA1*	824	15	15.0	35.6
	*DQB1*	988	16	11.9	34.0
	*DRB1*	901	33	11.1	32.3
	*DPA1*	982	4	78.7	37.0
	*DPB1*	875	20	28.9	36.8
Gene set	Class I	932	NA	2.6	36.1
	Classical 6	690	NA	1.2	35.0
	Classical 8	593	NA	0.5	35.5

*NA* not applicable

^1^1000 samples were available for typing at each gene

^2^Total number of 2-field alleles typed at each gene

^3^Coverage was calculated as the part of the HLA-VBSeq threshold

**Fig. 3:**
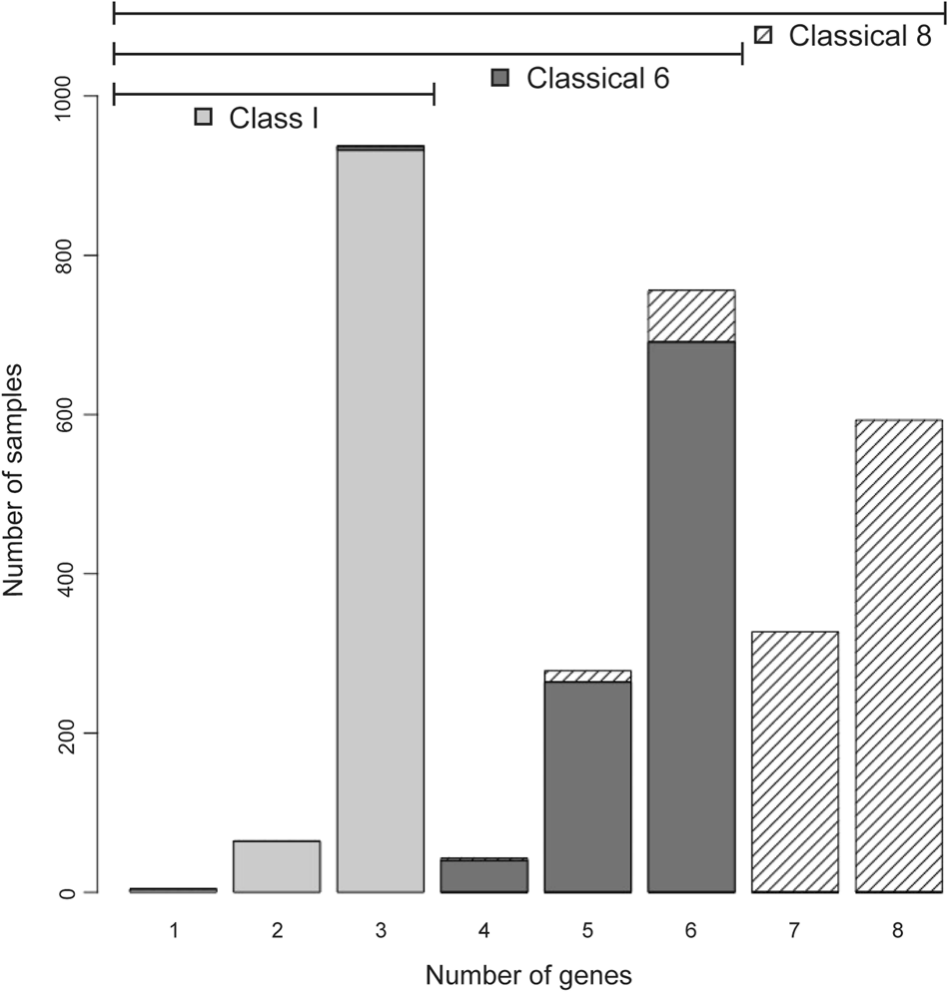
Distribution of samples typed in each stage during the creation of the complete SweHLA set. The majority of the 1000 SweGen samples were successfully called at all three class I genes (light gray, 3 genes *n* = 932). Building up to the classical 6 genes, 690 samples were genotyped successfully at all loci (dark gray), which decreased slightly for the classical 8 (hashed, 8 genes *n* = 593).

For the SweHLA class I gene set, 932 samples were called for all three genes, 60 samples for two genes and only four samples had one gene typed (Fig. 3). A similar pattern emerged as we built up to the classical 8 through the classical 6 set. The poorer SweHLA calling at the *HLA-DQA1* locus impacted this set, for which 690 samples had genotypes for all six genes. For the classical 8 gene set, 593 samples were typed at all genes, while 920 samples have high confidence calls at seven or more genes (Fig. 3).

An average of 22 alleles were called for each of the eight genes investigated for SweHLA (range 4–36, Table 1). This was not related to the absolute number of alleles available per software, but rather to Swedish population diversity. For example, while between 298 and 9854 2nd-field alleles were available across the software tested (SNP2HLA and HLAscan respectively, Supplementary Table S5), only 1.9% (class I) and 4.1% (class II) of the total number of alleles available were common across all programs (Supplementary Fig. S1A–B). However, an intersection of the alleles called (Supplementary Table S5), revealed that for class I, 32.5% of alleles were typed in all software (37.7% in *n−1* programs) and for class II this fraction was 55.7% (72.1% for *n−1* programs, Supplementary Fig. S1C–D).

**Fig. 4:**
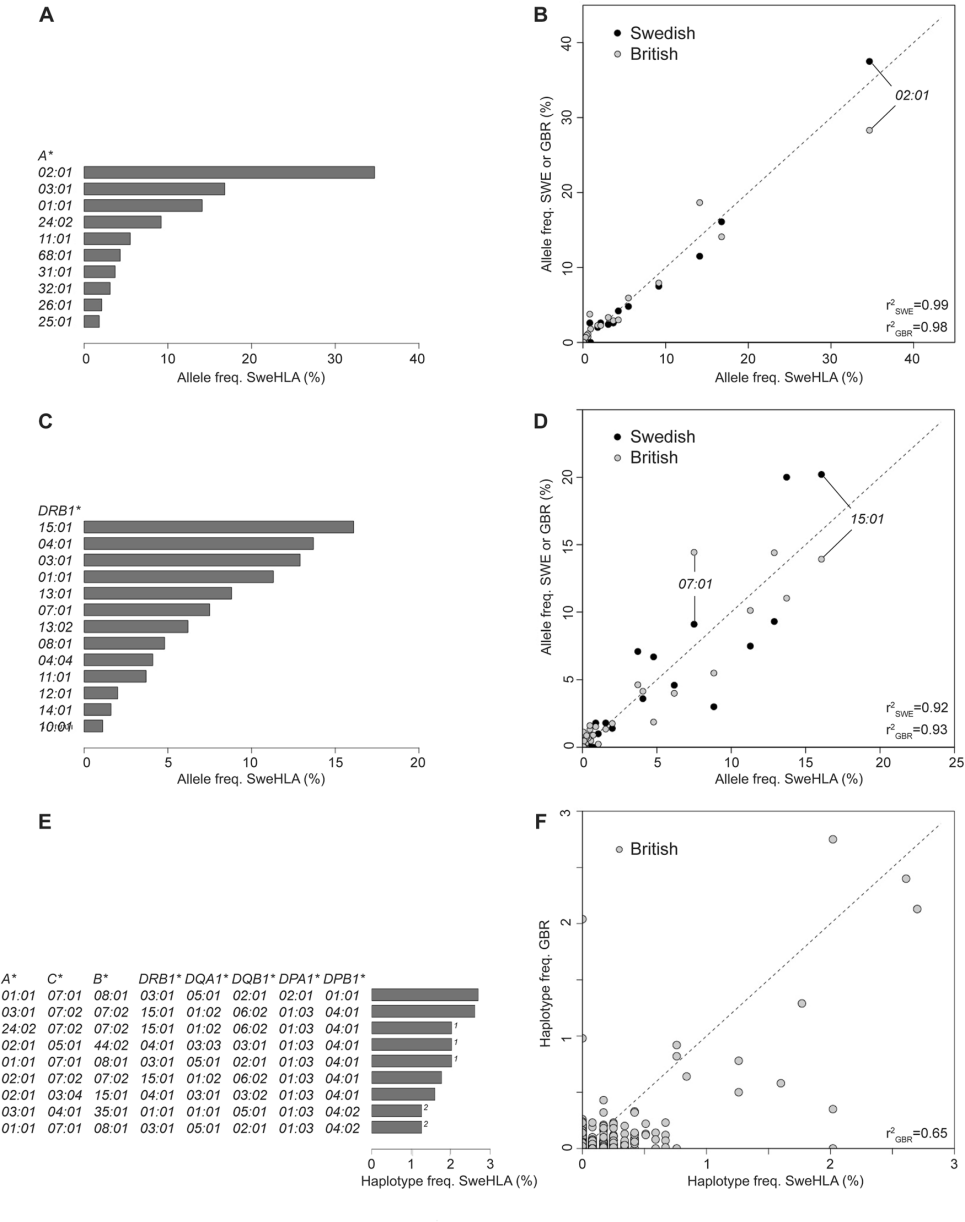
SweHLA allele frequency distribution and correlation (*r*^2^) to a separate lab typed Swedish population (SWE) and an imputed British set (GBR). **a**, **b** HLA-A*02:01 was the most frequent allele found in SweHLA, SWE, and GBR data sets, where as **c**, **d** the frequency pattern in *HLA-DRB1* varied across these three data sets. SweHLA alleles with frequencies above 1% are plotted in bar graphs. **e** The distribution of classical 8 allele haplotypes with a population frequency of <3% in SweHLA. (1) and (2) indicate haplotypes with the same allele frequency. **f** The correlation between SweHLA and GBR populations is reduced at the haplotype level.

We recorded population level frequencies for each gene and software combination (https://swefreq.nbis.se, 10.17044/NBIS/G000009) and noted that shared allele availability did not always translate to shared allele frequency. For example in class I genes, small frequency fluctuations were observed across data sets for *HLA-A* (A*26:01 ranged between 1.8–2.5%; 2.1% SweHLA, Fig. 4a), while larger discrepancies were noted for *HLA-B* (B*27:05: 4.8–8.0%; 7.6% in SweHLA, Supplementary Fig. S2A). In class II, the variations were larger and occurred more frequently. For *HLA-DRB1*, the most common allele HLA-DRB1*15:01, depending on software choice, the population allele frequency ranged from 6.1 to 17.7% (SweHLA frequency 16.1%, Fig. 4c).

In order to explore discrepancies more thoroughly, we plotted the concordance rate per allele against the frequency per allele for each gene and software (Supplementary Fig. S3). In general, SweHLA alleles observed at a frequency greater than 5% showed concordance above 90%. There were a few notable exceptions; HLA-B*27:05 and HLA-DRB1*15:01 as mentioned previously, as well as HLA-C*05:01, two *HLA-DPB1* and *HLA-DQA1* alleles (Supplementary Fig. S3B–E and H). In each case, the SweHLA allele frequency was 7.6% or above, with a concordance rate below 80%. We noted previously that *HLA-DQA1* had the lowest genotyping rate (824/1000 samples, Table 1) and Supplementary Fig. S3E illustrates that this problem is in large part due to missing reference data; seven alleles with a population allele frequency ranging 0.06–6.00% were not present in the SNP2HLA reference (dark blue line). Combined, these alleles represent 11% of *HLA-DQA1* diversity in SweHLA.

Given that SNP2HLA is an imputation program, we investigated if the original alignment of reads to the genome reference could have affected SNP availability for this process. This may indeed have been the case. There are eight curated European HLA haplotypes available for alignment, with GRCh37 incorporating the PGF haplotype [25] (Supplementary Table S2). This is important, as within exon 2 of *HLA-DQA1* there is a stretch of ~100 nucleotides, common to COX and QBL, but lacking in the other five haplotypes including PGF. Mapping to GRCh37 results in the soft clipping of reads which cannot align to the reference, and a dramatic drop in sequence coverage (Supplementary Fig. S4). The latter can affect allele calling in both homozygotes and heterozygotes, illustrated clearly when uncalled samples were aligned to either PGF, or alternate haplotypes (Supplementary Fig. S4).

### Allele and haplotype correlations across populations

The SweHLA population frequencies for *HLA-A*, *HLA-B*, *HLA-C*, *HLA-DQA1*, *HLA-DQB1*, and *HLA-DRB1* were highly correlated with those of an independent lab typed Swedish population (*r*^2^ spans between 0.87–0.99 for *HLA-DQA1* to *HLA-A*; black circles, Fig. 4b, d and Supplementary Fig. S5A, C and E). There was no evidence that the number of alleles typed influence the correlation. *HLA-DQA1* has 15 alleles and *HLA-DQB1* 16, yet *r*^2^ is 0.87 and 0.93, respectively. High levels of genetic homogeneity across HLA have been reported for Europe, with the diversity estimated to be as low as ~5% [26, 27]. It was therefore not surprising that the frequency comparison between SweHLA and >5500 British samples gave only slightly lower correlations than those to a Swedish population (*r*^2^ spans 0.83–0.98 for *HLA-DQA1* to *HLA-A*; gray circles, Fig. 4b, d and Supplementary Fig. S5).

Phased MHC blocks can be used in multiple downstream analyses, including the imputation of missing allele calls, creating population reference graphs, the investigation of allele group interactions and to dissect disease causing mechanisms [25, 28, 29]. In our phasing of the classical 8 gene haplotypes, only samples with complete allele typing per intermediate block were included. For example, block 1 (*HLA-C* and *HLA-B*) utilized the results of 948 samples, whereas block 2 (*HLA-DRB1, HLA-DQA1*, and *HLA-DQB1*) was reduced to 733 samples (Supplementary Fig. S6A–C). At the resolution of the classical 6 genes (Supplementary Fig. S6D), it was revealed that COX (7%) and PGF (4%) were the most common haplotypes in SweHLA. This result could be further teased apart at the classical 8 haplotype level (Fig. 4e), with PGF still among the most frequent at 2.6% of the total, however VAVY which shares the classical 6 haplotype with COX, became the most common haplotype (2.7%) [29].

The maximum haplotype frequency within the classical 6 data was below 7% and for the classical 8 it was reduced to below 3% (Fig. 4e, Supplementary Fig. S6D). This was in keeping with the frequencies reported for the British population [11]. Comparing across these populations, we can see the effect of recombination to shuffle common alleles to create rare haplotypes (classical 8 haplotype *r*^2^ = 0.648, Fig. 4f), however COX and PGF were also the most frequent 6 gene haplotypes in the British population, with VAVY ranked third in the 8 gene haplotype [11].

### Significant differences found in homozygosity rate

We explored the homozygosity of SweHLA (Table 1) in comparison to other European derived populations. Per gene, SweHLA was compared to European Americans (*HLA-A*, *HLA-B*, *HLA-C*, *HLA-DQA1*, and *HLA-DRB1)* [30] and for the classical 6 and 8 haplotypes SweHLA was compared with the same British population as before [11]. Both *HLA-A* and *HLA-B* were significantly more homozygous than European Americans (population proportional *Z*-score; 17.7% vs. 15.2%, *p-*value = 0.044 and 9.8% vs. 7.0%, *p-*value = 0.002, respectively). For the classical 6 and 8 haplotypes, no significant differences to the British cohort were observed (*p-*values: 0.44 and 0.31).

## Discussion

Drawn from the 1000 genomes of SweGen, SweHLA represents a high confidence bio-resource that provides a snapshot of Sweden’s MHC diversity. Data is reported at a clinically relevant resolution (2nd-field) and through the application of an *n−1* software concordance approach (Fig. 1), is expected to have minimal allele bias. Results for the classical 8 genes are available at both the allele and haplotype level, and so SweHLA could be used to estimate HLA diversity within this population, or to tease apart the patterns of linkage disequilibrium surrounding these genes. As SweHLA is drawn from SweGen, and therefore also the Swedish Twin Registry, the resource’s value likely lies as an added control resource for the genetic dissection of disease linked to HLA genes. Access to raw genotyping or phenotypic data (sex, age, and cohort) can be requested from each data set mentioned following an individual review process.

SweHLA is a consensus resource; for allele calls to be reported, three out of four software matches were required for class I genes, relaxed to two out of three for class II genes. The absolute number of HLA typing programs considered was arbitrary, but reflected a range of factors that end users of all software should take into account, (i) not all programs are developed to call the same gene set, (ii) the IMGT/HLA allele reference employed may be fixed or dated, (iii) algorithms differ between software.

Point one can be overcome through the selection of software to suit a specific need; although there is a lack of solutions developed to call outside class I, let alone the classical 8 genes set or those in the extended MHC region. Points two and three are perhaps the most clinically relevant, and reinforce the need for using multiple programs. Between 298 and 9854 2nd-field reference alleles were available for the software programs we used, however only a small fraction of these were common to all (1.9% class I and 4.1% class II). While this fraction increased in our population after typing (32.5% class I and 55.7% class II), the choice of IMGT/HLA reference could lead to incorrect assumptions. For example, HLA-DQA1*03:03 has a population frequency of 6% in SweHLA, but was not reported in the British population we used for comparison (Supplementary Fig. S5C). This was not a reflection of diversity, rather the fact that HLA-DQA1*03:03 is not available in the software used to analyse the British data set (Supplementary Fig. S3E). The flip side of this is when reference alleles are available, but not called due to the software’s algorithm. Here we use the example of HLA-DRB1*16:01, an allele previously associated with immune response in multiple sclerosis [31]. In our hands, HLA-DRB1*16:01 was called at a frequency of 0, 0.3, and 10.7% depending on software choice (Supplementary Fig. S3D). It may therefore appear that a locus is not replicated, rather than misestimated.

These concerns were not limited to class II alleles. For HLA-B*27:05, an allele with high association to several diseases, including ankylosing spondylitis [11, 32], we recorded allele frequency between 4.8 and 8.0%. Troublingly, one of the software had a concordance rate below 80% when compared with SweHLA (Supplementary Fig. S3B). Others have noted that the HLA-B*27 serotype has a higher frequency in the Nordic countries compared with other regions (>10% of *HLA-B* diversity [10, 33]), and so depending on population, this allele may appear rare (<5%) when in fact that is not the true case. These are not isolated examples. We noted 18 alleles with a delta allele frequency between any two HLA programs of more than 2%; 15 of these had reported association to at least one disease (Supplementary Table S6).

The problem of variant calling from reads aligned to regions of high genetic diversity, high repeat content or containing paralogous genes is not new [34, 35]. A clear bias toward calling reference alleles was noted when HLA SNPs genotyped from 1000G (phase I) short read NGS were compared to those generated for the same individuals via Sanger sequencing [36]. This trend was found in four of the five HLA genes examined, *HLA-A*, *HLA-B*, *HLA-DQB1*, and *HLA-C*, but not *HLA-DRB1* [36]. It is here that population reference graphs [29, 37] or alignment to the most similar MHC reference could aid variation discovery. We tested this at the *HLA-DQA1* locus with a subset of samples, typed or missing from SweHLA. First, we used the surrounding HLA classical 8 alleles to determine the most similar GRCh37 alternative haplotypes [29], and then aligned raw SweGen reads to those references and compared observable allelic variation. Supplementary Fig. S4 illustrates how the problem of soft clipping in exon 2 can be resolved for homozygotes (e.g., SweGen_A) and some heterozygotes (e.g., SweGen_B), but the problem is more challenging for heterozygotes for which the alternate extended haplotype is not yet available (e.g., SweGen_C). To overcome these issues, the community is developing software solutions using HLA population graphs as the reference. These aim not only to improve HLA inference, but also identify novel alleles (e.g., HLA*PRG [38] and Kourami [39]). However these provide G-group resolution [8], clustering HLA alleles together based on identical sequence at the peptide biding domain.

While it was not the aim of this project to identify novel HLA alleles, we nonetheless examined the genetic diversity across the MHC for this population (Fig. 2). Our results matched expectation, with the highest levels of Tajima’s D over the class I and II genes, and with more nucleotide diversity observed at class II genes compared to class I [40, 41]. The majority of SNP and indel variation fell into the 0–0.1 minor allele frequency bins. While a proportion of this will be true variation, as was noted above and by others, when the short read sequences are aligned to a more similar reference, a fraction of this variation will be revealed to be mapping errors [42].

With this work we have added to the growing set of HLA population resources now available for biomedicine. Whether the goal is to assess allele prevalence, dissect haplotype structure or develop a panel of additional control samples, the 1000 genomes sourced to build SweHLA will be extremely valuable. Here the development of an *n−1* high concordance HLA set cleanly illustrates the need to apply more than one program to the problem of calling MHC alleles from short read data sets.

## Supplementary information

Supplemental information

## Data Availability

The SweHLA population allele frequency data is available from the website https://swefreq.nbis.se (10.17044/NBIS/G000009). Flat files containing per individual HLA genotyping data generated from each software program, and for the final SweHLA data set, are available upon registration and agreement to terms and conditions for data download.
