# Supplementary material for: SweHLA: the high confidence HLA typing bio-resource drawn from 1000 Swedish genomes
Source: Eur J Hum Genet. 2019 Dec 16;28(5):627–35. doi: 10.1038/s41431-019-0559-2 (PMC7170882; doi:10.1038/s41431-019-0559-2)
Supplement: Supplementary file 1 — Supplemental information [file 41431_2019_559_MOESM1_ESM.docx]

**Supplementary Figures and Tables**

Supplementary Table S1. A subset of known HLA alleles defined based on reference sequence. 2

Supplementary Table S2. A subset of known HLA haplotypes from sequenced cell lines. 3

Supplementary Table S3. Distribution of known and novel variation across the MHC. 4

Supplementary Table S4. Number of samples that were HLA typed by each software 5

Supplementary Table S5. Summary of the number of alleles typed and the number available for typing within each software. 6

Supplementary Table S6. Examples of alleles implicated in disease for which the population frequency differences between software typing results. 7

Supplementary Figure S1. Intersection of available 15

Supplementary Figure S2. SweHLA allele frequencies. 16

Supplementary Figure S3. Illustration of the relationship between allele frequency called per software and the high confidence SweHLA set. 17

Supplementary Figure S4. Exon 2 for HLA-DQA1 is used to illustrate how soft clipping is resolved when a sample is aligned to the most similar alternate reference sequence. 18

Supplementary Figure S5. Between population correlations for six of the classical 8 genes. 19

# Supplementary Table S1. A subset of known HLA alleles defined based on reference sequence.

| HLA Allele | Nucleotide changes in exons 1-3^1^ |
| --- | --- |
| *A*26:01* | NM_002116.7:c.[28C>G; 98T>A; 102A>C; 257A>G; 259G>A; 261G>C; 362T>G; 363A>G; 413G>A; 423C>T; 517G>A; 539T>G; 555T>G; 559A>C; 560C>G] |
| *B*27:05* | NG_023187.1:c.[5T>G; 11T>C; 25G>C; 41C>G; 44C>G; 48C>A; 49C>G; 72C>T; 97T>C; 142T>A; 144A>C; 165C>G; 167A>T; 259A>G; 261C>G; 272A>G; 280C>A; 301A>G; 302G>A; 311A>C; 317G>T; 319G>C; 362G>A; 363C>T; 369C>T; 409C>T; 411T>C; 412G>C; 418T>G; 463C>A; 527A>T; 539G>T; 603C>G; 605A>C; 610G>C; 618T>G] |
| *DRB1*15:01* | NM_002124.3 |
| *C*05:01* | NM_002117.5:c.[22G>A; 28C>A; 47G>C; 97G>T; 142T>G; 176G>A; 201G>A; 289G>A; 302G>A; 312C>A; 341A>C; 368C>A; 412G>A; 419C>T; 453C>T; 485C>A; 486C>G; 512T>G; 527C>A; 539T>G; 601G>A; 618A>G] |
| *DQA1*03:03* | NM_002122.3:c.[22C>A; 39T>C; 60C>T; 90C>T; 101G>A; 102T>C; 122T>C; 126C>T; 138C>G; 146C>G; 165T>C; 169C>G; 177C>T; 203C>T; 209G>A; 212G>T; 217G>C; 218A>T; 223A>C; 227A>G; 232G>A; 235G>A; 237T>A; 249G>A; 250G>T; 251G>T; 260G>C; 267G>C; 274G>C; 275C>T; 282C>T; 295A>G; 308A>C; 396T>C; 438A>C; 456G>C; 548C>A; 591C>T; 592C>G] |
| *DRB1*16:01* | NM_002124.3:c.[85T>G; 227T>A; 286A>T; 294G>A; 295C>G; 297G>C; 298G>A; 299C>G; 303G>C; 344T>G; 345G>T] |

^1^The HGVS nomenclature for all alleles is maintained at https://www.ebi.ac.uk/ipd/imgt/hla/allele.html

# Supplementary Table S2. A subset of known HLA haplotypes from sequenced cell lines.

| IHWG cell line^1^ | GRCh37^2^ | HLA Allele^3^ | | | | | | | |
| --- | --- | --- | --- | --- | --- | --- | --- | --- | --- |
| PGF | chr6 | *A*03:01* | *B*07:02* | *C*07:02* | *DRB1*15:01* | *DQA1*01:02* | *DQB1*06:02* | *DPA1*01:03* | *DPB1*04:01* |
| COX | chr6_cox_hap2 | *A*01:01* | *B*08:01* | *C*07:01* | *DRB1*03:01* | *DQA1*05:01* | *DQB1*02:01* | *DPA1*01:03* | *DPB1*03:01* |
| DBB | chr6_dbb_hap3 | *A*02:01* | *B*57:01* | *C*06:02* | *DRB1*07:01* | *DQA1*02:01* | *DQB1*03:03* | *DPA1*01:03* | *DPB1*04:01* |
| MANN | chr6_mann_hap4 | *A*29:02* | *B*44:03* | *C*16:01* | *DRB1*07:01* | *DQA1*02:01* | *DQB1*02:02* | *DPA1*01:03* | *DPB1*02:01* |
| MCF | chr6_mcf_hap5 | *A*02:01* | *B*15:01* | *C*03:04* | *DRB1*04:01* | *DQA1*03:03* | *DQB1*03:01* | *DPA1*01:03* | *DPB1*04:02* |
| QBL | chr6_qbl_hap6 | *A*26:01* | *B*18:01* | *C*05:01* | *DRB1*03:01* | *DQA1*05:01* | *DQB1*02:01* | *DPA1*01:03* | *DPB1*02:02* |
| SSTO | chr6_ssto_hap7 | *A*32:01* | *B*44:02* | *C*05:01* | *DRB1*04:03* | *DQA1*03:01* | *DQB1*03:05* | *DPA1*01:03* | *DPB1*04:01* |
| VAVY | NA | *A*01:01* | *B*08:01* | *C*07:01* | *DRB1*03:01* | *DQA1*05:01* | *DQB1*02:01* | *DPA1*02:01* | *DPB1*01:01* |

^1^Cell line name as per the International Histocompatibility Working Group. ^2^Name given to each haplotype within the GRCH37 reference genome. ^3^Two-field resolution haplotypes of selected haplotypes adapted from IHWG [https://www.ebi.ac.uk] and [doi:10.1101/gr.213538.116]. NA, not applicable. The HGVS nomenclature for all alleles is maintained at https://www.ebi.ac.uk/ipd/imgt/hla/allele.html.

# Supplementary Table S3. Distribution of known and novel variation across the MHC.

| Variant type | Bin^1^ | rs^2^ | non-rs^2^ | % novel |
| --- | --- | --- | --- | --- |
| Indel | 0.0 - 0.1 | 3720 | 7176 | 65.6 |
|  | 0.1 - 0.2 | 849 | 578 | 40.5 |
|  | 0.2 - 0.3 | 634 | 383 | 37.7 |
|  | 0.3 - 0.4 | 424 | 241 | 36.2 |
|  | 0.4 - 0.5 | 344 | 156 | 31.2 |
|  | total | 5971 | 8534 | 58.8 |
| SNP | 0.0 - 0.1 | 29038 | 18804 | 39.3 |
|  | 0.1 - 0.2 | 8645 | 202 | 2.3 |
|  | 0.2 - 0.3 | 6879 | 211 | 3.0 |
|  | 0.3 - 0.4 | 4982 | 150 | 2.9 |
|  | 0.4 - 0.5 | 4158 | 63 | 1.5 |
|  | total | 53702 | 19430 | 26.6 |

^1^Bins represent minor allele frequency (MAF) steps of 0.1 or the total content of MHC region. ^2^rs designations are from dbSNP v147 as per SweGen [doi:10.1038/ejhg.2017.130].

# Supplementary Table S4. Number of samples that were HLA typed by each software

| Gene or gene set | HLAscan | HLA-VBSeq | SNP2HLA | OptiType |
| --- | --- | --- | --- | --- |
| A | 1000 | 992 | 999 | 1000 |
| *B* | 1000 | 988 | 1000 | 1000 |
| *C* | 1000 | 986 | 1000 | 1000 |
| *DQA1* | 993 | 1000 | 998 | NA |
| *DQB1* | 987 | 1000 | 1000 | NA |
| *DRB1* | 971 | 999 | 1000 | NA |
| *DPA1* | 938 | 1000 | 1000 | NA |
| *DPB1* | 959 | 1000 | 1000 | NA |
| Class I MHC genes | 1000 | 968 | 999 | 1000 |
| Classical 6 | 951 | 967 | 994 | NA |
| Classical 8 | 854 | 967 | 997 | NA |

Maximum number of samples available, 1000. NA, not applicable.

# Supplementary Table S5. Summary of the number of alleles typed and the number available for typing within each software.

|  | Software^1^ | | | |
| --- | --- | --- | --- | --- |
| Gene | HLAscan | HLA-VBSeq | SNP2HLA | OptiType |
| *A* | 40 (2382) | 62 (947) | 32 (50) | 28 (1678) |
| *B* | 52 (3048) | 48 (1053) | 42 (97) | 46 (2273) |
| *C* | 34 (2026) | 64 (1077) | 22 (33) | 25 (1311) |
| *DPA1* | 4 (20) | 4 (13) | 5 (7) | NA (NA) |
| *DPB1* | 22 (462) | 30 (127) | 21 (34) | NA (NA) |
| *DQA1* | 21 (33) | 15 (26) | 8 (8) | NA (NA) |
| *DQB1* | 17 (559) | 21 (112) | 16 (18) | NA (NA) |
| *DRB1* | 37 (1324) | 29 (46) | 35 (51) | NA (NA) |

^1^alleles typed (alleles available) per software and gene. NA, not applicable. Alleles at typed 2-field resolution

# Supplementary Table S6. Examples of alleles implicated in disease for which the population frequency differences between software typing results.

|  | Individual software program | | | |  |  |  |
| --- | --- | --- | --- | --- | --- | --- | --- |
| Alleles | HLA-VBSeq | HLAscan | SNP2HLA | OptiType | SweHLA | Published disease association | |
| *A*26:01* | 0.018 | 0.024 | 0.020 | 0.025 | 0.021 | Behcet's disease^1^, Vitiligo^2^, Idiopathic Hypoparathyroidism^3^ |  |
| *A*32:01* | 0.017 | 0.030 | 0.031 | 0.031 | 0.031 | Vancomycin-induced drug reaction^4^, T1D^5^ |  |
| *B*27:05* | 0.080 | 0.048 | 0.075 | 0.078 | 0.076 | AS^6,7^, SpA^8^, Rapid HIV disease progression^9^, PA^10,11^, juvenile idiopathic arthritis ^6^ |  |
| *B*39:01* | 0.015 | 0.011 | 0.004 | 0.012 | 0.010 | T1D^12,13^, PA^10^, HIV-1^14^, Familial Mediterranean Fever^15^ |  |
| *C*05:01* | 0.094 | 0.060 | 0.093 | 0.093 | 0.090 | PA^11^ |  |
| *C*15:02* | 0.001 | 0.020 | 0.021 | 0.021 | 0.019 | MS^16^ |  |
| *DPA1*01:03* | 0.873 | 0.902 | 0.863 | NA | 0.880 | Narcolepsy^17^, Leukemia^18^, AS^19,20^, inflammatory bowel diseases^21^ |  |
| *DPB1*04:01* | 0.433 | 0.486 | 0.471 | NA | 0.491 | Non-obstructive azoospermia^22^, Narcolepsy^17^, Scleroderma^23^ |  |
| *DQA1*01:04* | 0.025 | 0.028 | NA | NA | 0.002 | T1D^24–27^, Takayasu arteritis^28^ |  |
| *DQA1*03:01* | 0.119 | 0.094 | 0.208 | NA | 0.135 | T1D^24–27^, Takayasu arteritis^28^ |  |
| *DQA1*03:03* | 0.077 | 0.075 | NA | NA | 0.060 | T1D^24–27^, Takayasu arteritis^28^ |  |
| *DQA1*05:01* | 0.121 | 0.129 | 0.199 | NA | 0.127 | T1D^24,25^, BP^29^, CD^30,31^ |  |
| *DQA1*05:05* | 0.077 | 0.028 | NA | NA | 0.027 | BP^32^, CD^31^ |  |
| *DQB1*02:02* | 0.009 | 0.045 | 0.043 | NA | 0.041 | CD^33^, T1D^34^, Asparaginase Hypersensitivity In Acute Lymphoblastic Leukemia^35^ |  |
| *DRB1*04:03* | 0.043 | 0.004 | 0.002 | NA | 0.002 | Crohn’s disease^36^, SLE^37^, oxcarbazepine‐induced maculopapular eruption^38^, Cervical Carcinoma^39^ |  |
| *DRB1*04:04* | 0.003 | 0.042 | 0.044 | NA | 0.041 | MS^40^, RA^24,41^ |  |
| *DRB1*08:01* | NA | 0.062 | 0.064 | NA | 0.048 | SLE^42^, RA^43,44^, BP^29^, T1D^13^, |  |
| *DRB1*08:02* | 0.067 | 0.002 | 0.000 | NA | 0.002 | SLE^37^ |  |
| *DRB1*14:01* | 0.002 | 0.017 | 0.021 | NA | 0.016 | Sarcoidosis^45^ |  |
| *DRB1*15:01* | 0.175 | 0.169 | 0.061 | NA | 0.161 | SLE^37^, MS^46^ |  |
| *DRB1*16:01* | 0.000 | 0.003 | 0.107 | NA | 0.003 | MS^47^ |  |

SLE= Systemic Lupus Erythematosus, MS= Multiple Sclerosis, AS=Ankylosing spondylitis, RA=Rheumatoid arthritis, T1D=type 1 diabetes, BP=Bullous Pemphigoid, PA= psoriatic arthritis, CD=Celiac disease, SpA= Spondyloarthritis

1 Kang EH, Kim JY, Takeuchi F *et al.* Associations between the HLA-A polymorphism and the clinical manifestations of Behcet’s disease. *Arthritis Res Ther* 2011; **13**: R49.

2 Singh A, Sharma P, Kar HK *et al.* HLA Alleles and Amino-Acid Signatures of the Peptide-Binding Pockets of HLA Molecules in Vitiligo. *J Invest Dermatol* 2012; **132**: 124–134.

3 Goswami R, Singh A, Gupta N, Indian Genome Variation Consortium R, Rani R. Presence of strong association of the major histocompatibility complex (MHC) class I allele HLA-A*26:01 with idiopathic hypoparathyroidism. *J Clin Endocrinol Metab* 2012; **97**: E1820-4.

4 Konvinse KC, Trubiano JA, Pavlos R *et al.* HLA-A*32:01 is strongly associated with vancomycin-induced drug reaction with eosinophilia and systemic symptoms. *J Allergy Clin Immunol* 2019; **144**: 183–192.

5 Noble JA, Valdes AM. Genetics of the HLA region in the prediction of type 1 diabetes. *Curr Diab Rep* 2011; **11**: 533–42.

6 Srivastava R, Agnihotry S, Aggarwal R, Bajpai P, Aggarwal A. HLA-B27 subtypes in enthesitis-related arthritis category of juvenile idiopathic arthritis and ankylosing spondylitis in northern India. *Clin Exp Rheumatol*; **33**: 931–5.

7 Loll B, Fabian H, Huser H *et al.* Increased conformational flexibility characterizes HLA-B*27 subtypes associated with ankylosing spondylitis. *Arthritis Rheumatol* 2015; **68**: n/a-n/a.

8 Gómez P, Montserrat V, Marcilla M, Paradela A, de Castro JAL. B*2707 differs in peptide specificity from B*2705 and B*2704 as much as from HLA-B27 subtypes not associated to spondyloarthritis. *Eur J Immunol* 2006; **36**: 1867–81.

9 Brener J, Gall A, Hurst J *et al.* Rapid HIV disease progression following superinfection in an HLA-B*27:05/B*57:01-positive transmission recipient. *Retrovirology* 2018; **15**: 7.

10 Winchester R, Minevich G, Steshenko V *et al.* HLA associations reveal genetic heterogeneity in psoriatic arthritis and in the psoriasis phenotype. *Arthritis Rheum* 2012; **64**: 1134–1144.

11 Haroon M, Winchester R, Giles JT, Heffernan E, FitzGerald O. Certain class I HLA alleles and haplotypes implicated in susceptibility play a role in determining specific features of the psoriatic arthritis phenotype. *Ann Rheum Dis* 2016; **75**: 155–62.

12 Mikk M-L, Heikkinen T, El-Amir MI *et al.* The association of the HLA-A*24:02, B*39:01 and B*39:06 alleles with type 1 diabetes is restricted to specific HLA-DR/DQ haplotypes in Finns. *HLA* 2017; **89**: 215–224.

13 Mikk M-L, Kiviniemi M, Laine A-P *et al.* The HLA-B*39 allele increases type 1 diabetes risk conferred by HLA-DRB1*04:04-DQB1*03:02 and HLA-DRB1*08-DQB1*04 class II haplotypes. *Hum Immunol* 2014; **75**: 65–70.

14 Valenzuela-Ponce H, Alva-Hernández S, Garrido-Rodríguez D *et al.* Novel HLA class I associations with HIV-1 control in a unique genetically admixed population. *Sci Rep* 2018; **8**: 6111.

15 Yasunami M, Nakamura H, Agematsu K *et al.* Identification of Disease-Promoting HLA Class I and Protective Class II Modifiers in Japanese Patients with Familial Mediterranean Fever. *PLoS One* 2015; **10**: e0125938.

16 Cree BAC, Rioux JD, McCauley JL *et al.* A major histocompatibility Class I locus contributes to multiple sclerosis susceptibility independently from HLA-DRB1*15:01. *PLoS One* 2010; **5**: e11296.

17 Ollila HM, Ravel J-M, Han F *et al.* HLA-DPB1 and HLA Class I Confer Risk of and Protection from Narcolepsy. *Am J Hum Genet* 2015; **96**: 136–146.

18 Rivera-Pirela SE, Echeverría M, Salcedo P *et al.* [HLA DRB1*, DQB1*, DPA1*, and DPB1* and their association with the pathogenesis of leukemia in the population of Venezuela]. *Rev Alerg Mex* 2016; **63**: 237–51.

19 Díaz-Peña R, Aransay AM, Bruges-Armas J *et al.* Fine mapping of a major histocompatibility complex in ankylosing spondylitis: Association of the HLA-DPA1 and HLA-DPB1 regions. *Arthritis Rheum* 2011; **63**: 3305–3312.

20 Díaz-Peña R, Castro-Santos P, Aransay AM, Brüges-Armas J, Pimentel-Santos FM, López-Larrea C. Genetic study confirms association of HLA-DPA1∗01:03 subtype with ankylosing spondylitis in HLA-B27-positive populations. *Hum Immunol* 2013; **74**: 764–767.

21 Goyette P, Boucher G, Mallon D *et al.* High-density mapping of the MHC identifies a shared role for HLA-DRB1*01:03 in inflammatory bowel diseases and heterozygous advantage in ulcerative colitis. *Nat Genet* 2015; **47**: 172–179.

22 Jinam TA, Nakaoka H, Hosomichi K *et al.* HLA-DPB1*04:01 allele is associated with non-obstructive azoospermia in Japanese patients. *Hum Genet* 2013; **132**: 1405–11.

23 Wang J, Guo X, Yi L *et al.* Association of HLA-DPB1 with scleroderma and its clinical features in Chinese population. *PLoS One* 2014; **9**: e87363.

24 Caillat-Zucman S. New insights into the understanding of MHC associations with immune-mediated disorders. *HLA* 2017; **89**: 3–13.

25 Onengut-Gumuscu S, Chen W-M, Robertson CC *et al.* Type 1 Diabetes Risk in African-Ancestry Participants and Utility of an Ancestry-Specific Genetic Risk Score. *Diabetes Care* 2019; **42**: 406–415.

26 Howson JMM, Roy MS, Zeitels L, Stevens H, Todd JA. HLA class II gene associations in African American type 1 diabetes reveal a protective HLA-DRB1*03 haplotype. *Diabet Med* 2013; **30**: 710–6.

27 Hamzeh AR, Nair P, Al-Khaja N, Al Ali MT. Association of HLA-DQA1 and -DQB1 alleles with type I diabetes in Arabs: a meta-analyses. *Tissue Antigens* 2015; **86**: 21–27.

28 Lv N, Wang Z, Dang A *et al.* HLA-DQA1, DQB1 and DRB1 alleles associated with Takayasu arteritis in the Chinese Han population. *Hum Immunol* 2015; **76**: 241–4.

29 Esmaili N, Mortazavi H, Chams-Davatchi C *et al.* Association between HLA-DQB1*03:01 and Bullous pemphigoid in Iranian patients. *Iran J Immunol* 2013; **10**: 1–9.

30 Lenz TL, Deutsch AJ, Han B *et al.* Widespread non-additive and interaction effects within HLA loci modulate the risk of autoimmune diseases. *Nat Genet* 2015; **47**: 1085–1090.

31 Megiorni F, Pizzuti A. HLA-DQA1 and HLA-DQB1 in Celiac disease predisposition: practical implications of the HLA molecular typing. *J Biomed Sci* 2012; **19**: 88.

32 Chagury AA, Sennes LU, Gil JM *et al.* HLA-C*17, DQB1*03:01, DQA1*01:03 and DQA1*05:05 Alleles Associated to Bullous Pemphigoid in Brazilian Population. *Ann Dermatol* 2018; **30**: 8–12.

33 Karell K, Louka AS, Moodie SJ *et al.* Hla types in celiac disease patients not carrying the DQA1*05-DQB1*02 (DQ2) heterodimer: results from the european genetics cluster on celiac disease. *Hum Immunol* 2003; **64**: 469–477.

34 Mosaad YM, Auf FA, Metwally SS *et al.* HLA-DQB1* alleles and genetic susceptibility to type 1 diabetes mellitus. *World J Diabetes* 2012; **3**: 149–55.

35 Kutszegi N, Yang X, Gézsi A *et al.* HLA-DRB1*07:01-HLA-DQA1*02:01-HLA-DQB1*02:02 haplotype is associated with a high risk of asparaginase hypersensitivity in acute lymphoblastic leukemia. *Haematologica* 2017; **102**: 1578–1586.

36 Han B, Akiyama M, Kim K-K *et al.* Amino acid position 37 of HLA-DRβ1 affects susceptibility to Crohn’s disease in Asians. *Hum Mol Genet* 2018; **27**: 3901–3910.

37 Furukawa H, Kawasaki A, Oka S *et al.* Human Leukocyte Antigens and Systemic Lupus Erythematosus: A Protective Role for the HLA-DR6 Alleles DRB1*13:02 and *14:03. *PLoS One* 2014; **9**: e87792.

38 Moon J, Kim T-J, Lim J-A *et al.* HLA-B*40:02 and DRB1*04:03 are risk factors for oxcarbazepine-induced maculopapular eruption. *Epilepsia* 2016; **57**: 1879–1886.

39 Alaez-Verson C, Berumen-Campos J, Munguía-Saldaña A *et al.* HPV-16 and HLA-DRB1 alleles are associated with cervical carcinoma in Mexican Mestizo women. *Arch Med Res* 2011; **42**: 421–5.

40 Buck D, Cepok S, Hoffmann S *et al.* Influence of the HLA-DRB1 Genotype on Antibody Development to Interferon Beta in Multiple Sclerosis. *Arch Neurol* 2011; **68**: 480.

41 Seidl C, Körbitzer J, Badenhoop K *et al.* Protection against severe disease is conferred by DERAA-bearing HLA-DRB1 alleles among HLA-DQ3 and HLA-DQ5 positive rheumatoid arthritis patients. *Hum Immunol* 2001; **62**: 523–9.

42 Cruz GI, Shao X, Quach H *et al.* A Child’s HLA-DRB1 genotype increases maternal risk of systemic lupus erythematosus. *J Autoimmun* 2016; **74**: 201–207.

43 Roark CL, Anderson KM, Aubrey MT, Rosloniec EF, Freed BM. Arthritogenic peptide binding to DRB1*01 alleles correlates with susceptibility to rheumatoid arthritis. *J Autoimmun* 2016; **72**: 25–32.

44 Freed BM, Schuyler RP, Aubrey MT. Association of the HLA-DRB1 epitope LA(67, 74) with rheumatoid arthritis and citrullinated vimentin binding. *Arthritis Rheum* 2011; **63**: 3733–9.

45 Wennerström A, Pietinalho A, Vauhkonen H *et al.* HLA-DRB1 allele frequencies and C4 copy number variation in Finnish sarcoidosis patients and associations with disease prognosis. *Hum Immunol* 2012; **73**: 93–100.

46 Cocco E, Meloni A, Murru MR *et al.* Vitamin D Responsive Elements within the HLA-DRB1 Promoter Region in Sardinian Multiple Sclerosis Associated Alleles. *PLoS One* 2012; **7**: e41678.

47 Buck D, Cepok S, Hoffmann S *et al.* Influence of the HLA-DRB1 Genotype on Antibody Development to Interferon Beta in Multiple Sclerosis. *Arch Neurol* 2011; **68**: 480.


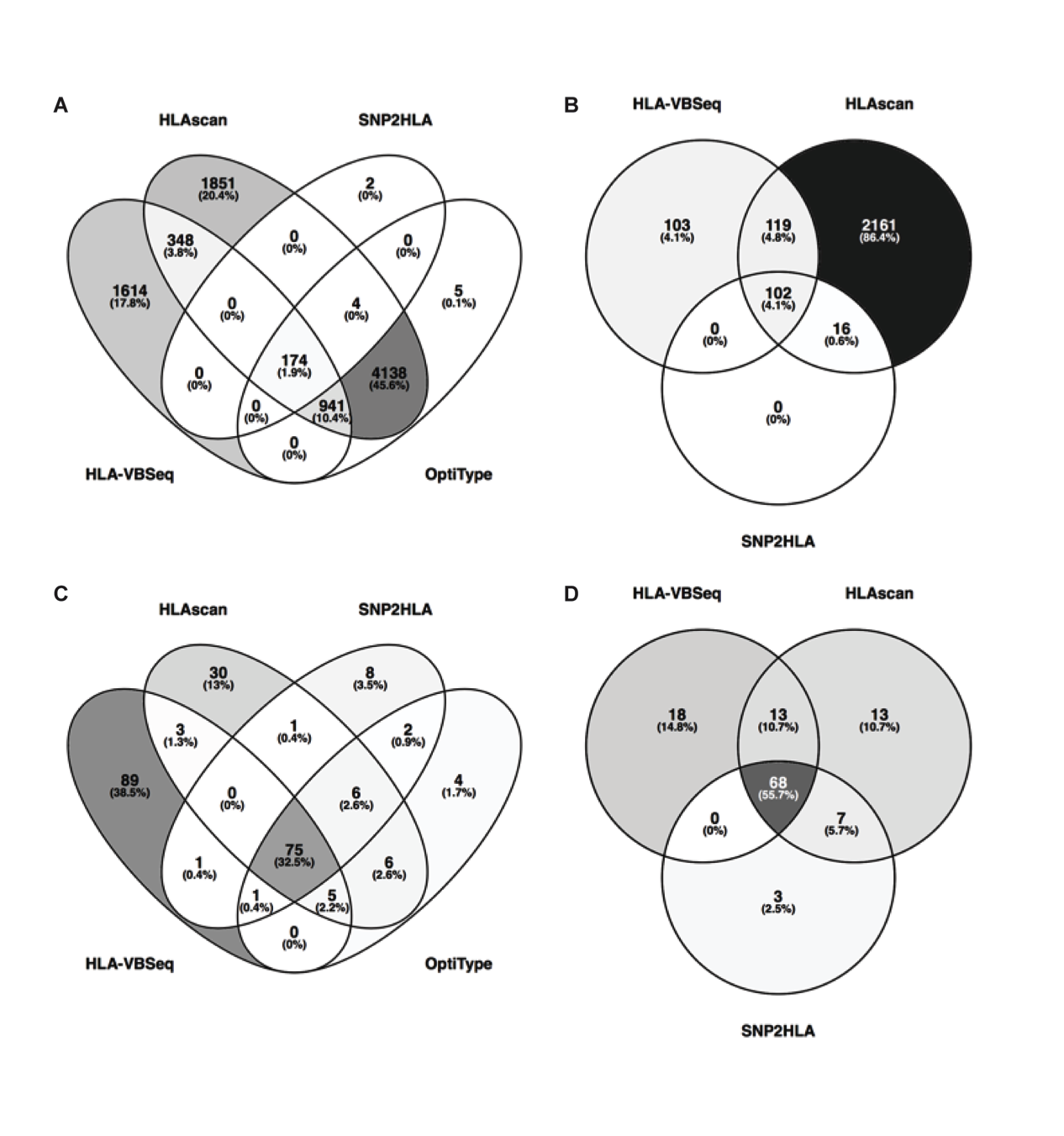


# Supplementary Figure S1. Intersection of available

A) class I and B) class II, and typed; C) class I and D) class II 2-field resolution reference alleles for each software.

#
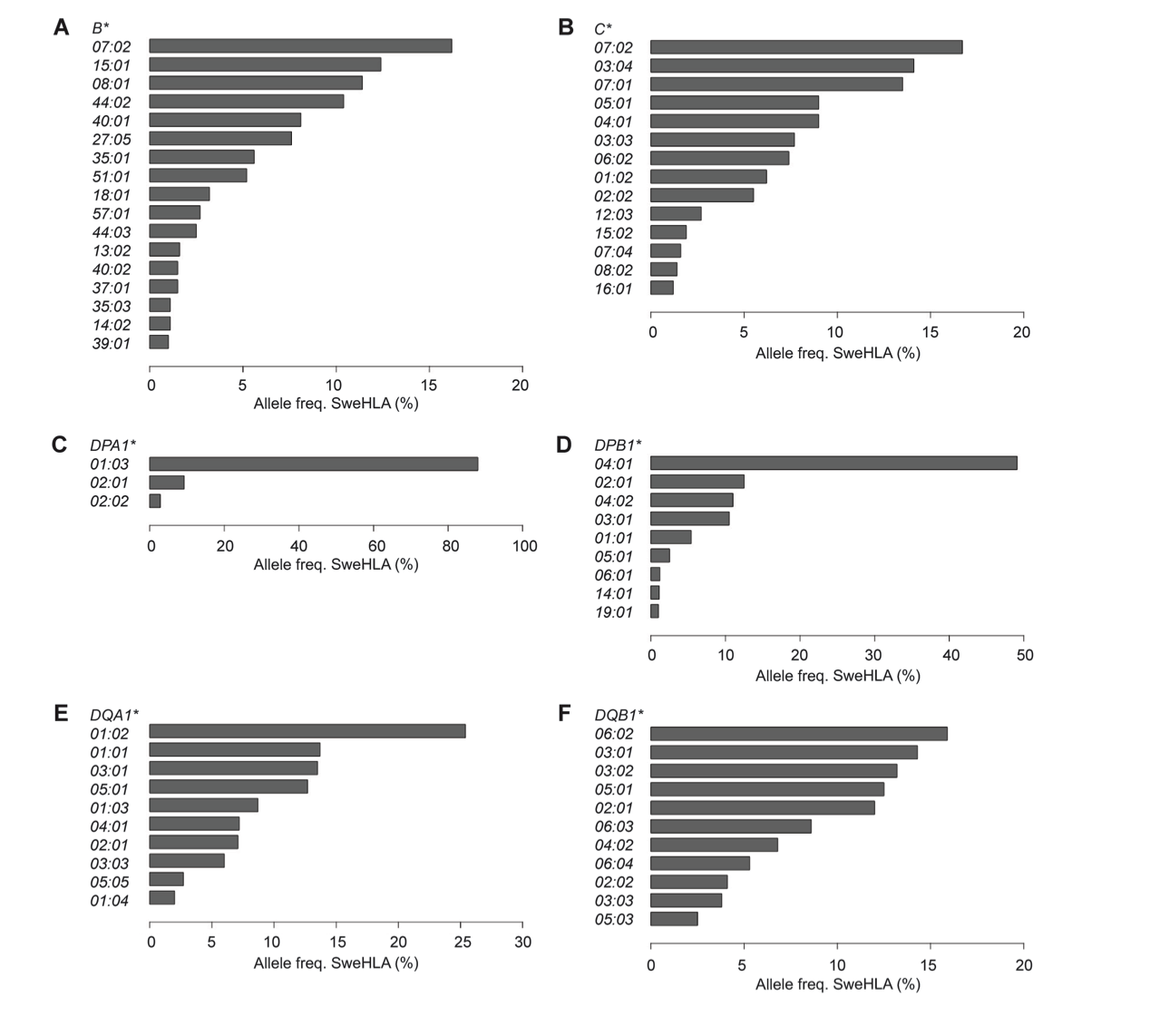


# Supplementary Figure S2. SweHLA allele frequencies.

(A) *HLA-B*, (B) *HLA-C*, (C) *HLA-DPA1*, (D) *HLA-DPB1*, (E) *HLA-DQA1* and (F) *HLA-DQB1*. Alleles with frequency greater than 1% are illustrated.

**
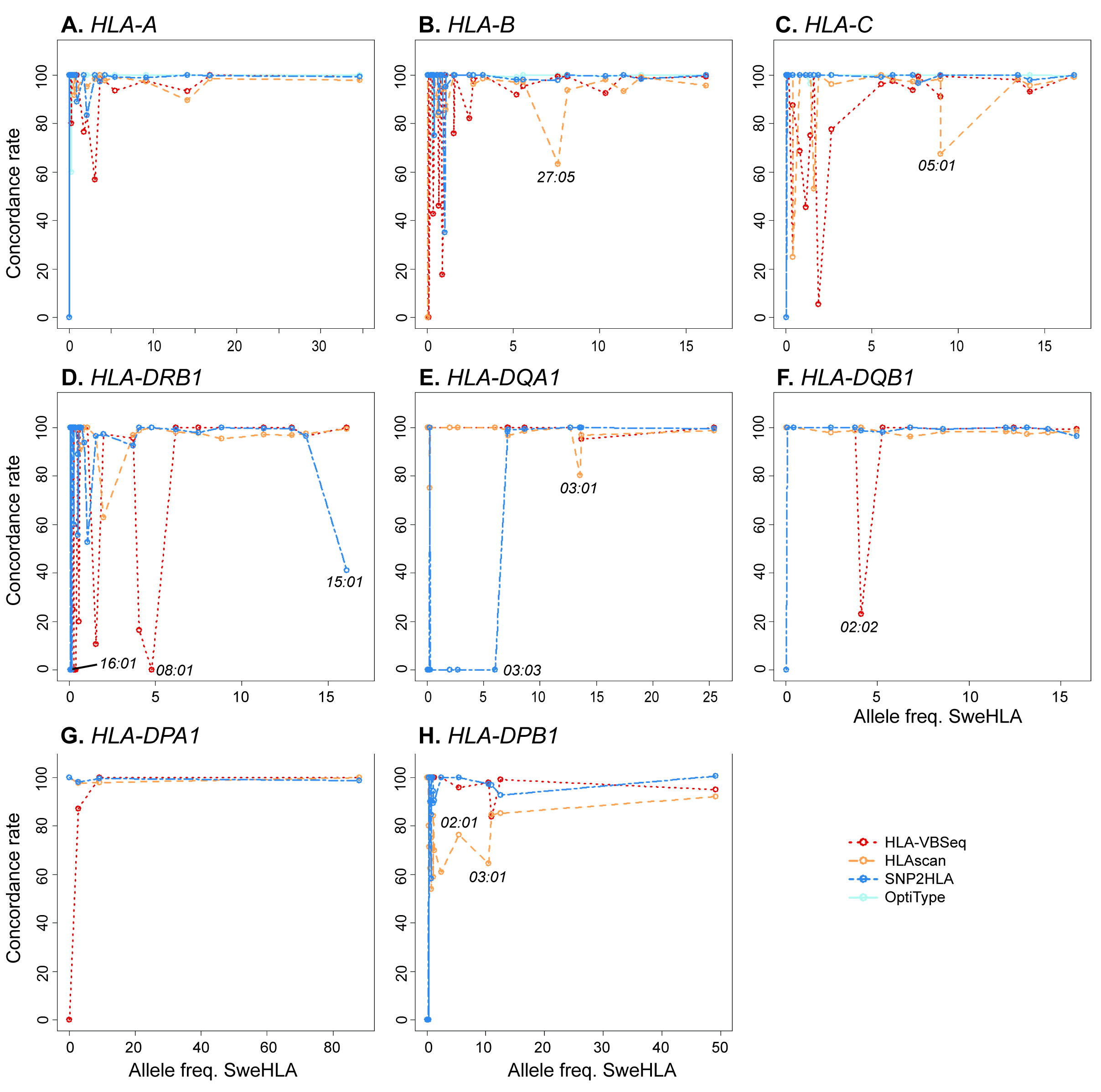
**

# Supplementary Figure S3. Illustration of the relationship between allele frequency called per software and the high confidence SweHLA set.

(A) *HLA-A*, (B) *HLA-B*, (C) *HLA-C*, (D) *HLA-DRB1*, (E) *HLA-DQA1,* (F) *HLA-DQB1*, (G) *HLA-DPA1* and (H) *HLA-DPB1*. Software used for genotyping were HLA-VBSeq (red), HLAscan (yellow), SNP2HLA (dark blue) and OptiType (light blue).

# Supplementary Figure S4. Exon 2 for HLA-DQA1 is used to illustrate how soft clipping is resolved when a sample is aligned to the most similar alternate reference sequence.

A region of soft clipping is indicated by a loss of read depth, as the DNA sequence from raw reads cannot be measured, as there is no reference sequence data to which they can align. For example, individual SweGen A was typed in the SweHLA set as homozygote *05:01/05:01*. The default MHC reference sequence for GRCh37 chr6 is from the IHWG cell line PGF (*01:02/01:02*, Supplementary Table S1). Read depth is recovered when this sample was aligned to the reference chr_cox_hap2 with the sample genotype. The heterozygote SweGen B is illustrated as well as the more complex case, SweGen C. The latter was not successfully genotyped in SweHLA (NA/NA), instead each software program resulted in a different genotypes (i.e. *02:01/01:01*, *01:01/01:01* or *01:01/03:01*). The pattern of soft clipping was reduced when the raw reads from this sample were aligned to the most similar reference sequence, chr6_ssto_hap7. Images are captured from IGV.


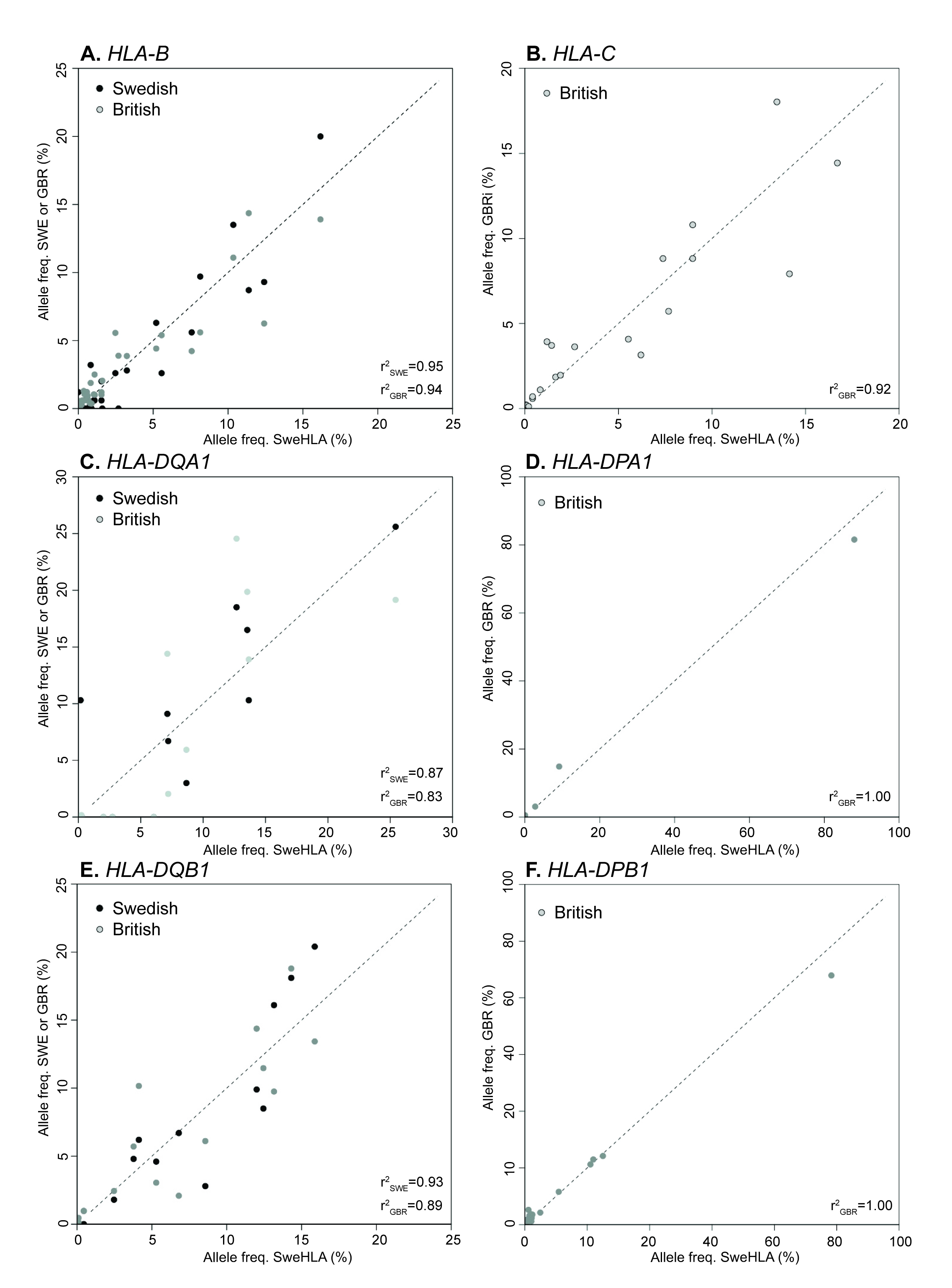


# Supplementary Figure S5. Between population correlations for six of the classical 8 genes.

Illustrated are SweHLA versus a lab typed Swedish cohort (SWE, black) and versus a British resource imputed with SNP2HLA (GBR, grey). (A) *HLA-B*, (B) *HLA-C*, (C) *HLA-DQA1*, (D) *HLA-DPA1*, (E) *HLA-DQB1,* (F) *HLA-DPB1*.


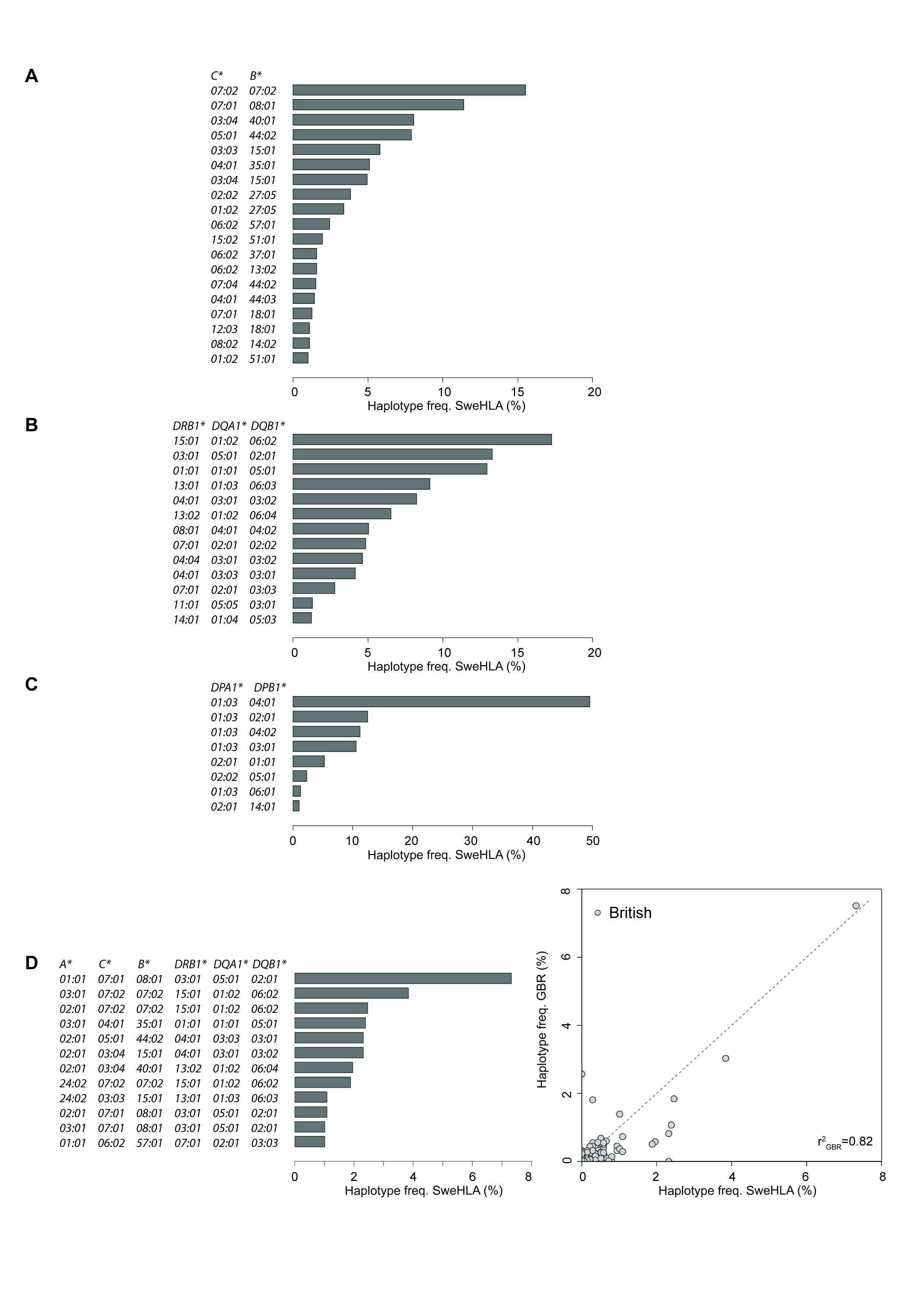
**Supplementary Figure S6. SweHLA haplotype frequencies at each of the three blocks used to construct the classical 8 gene haplotype.**

(A) Block 1, *HLA-B*, *-C*, (B) *HLA-DRB1, -DQA1, -DQB1* and (C) *HLA-DPA1, -DPB1*. (D) The SweHLA haplotypes frequencies for the classical 6 genes are plotted, as is the correlation between these and the British resource imputed with SNP2HLA (GBR, grey). Frequencies above 1% are plotted in bar graphs.
